# The role of the gastrocnemius flap in implant retention strategies for acute periprosthetic joint infection following total knee arthroplasty: a systematic review

**DOI:** 10.1007/s00402-026-06199-2

**Published:** 2026-01-28

**Authors:** Daniele Grassa, Guido Bocchino, Cesare Stefanelli, Giacomo Capece, Rocco Maria Comodo, Alessandro El Motassime, Riccardo Totti, Giulio Maccauro, Raffaele Vitiello

**Affiliations:** 1 Department of Orthopedics and Geriatric Sciences, Catholic University of the Sacred Heart, Roma, Italy; 2 Department of Orthopedics, Ageing and Rheumatological Sciences, Agostino Gemelli University Polyclinic, Rome, Italy; 3 Institute of Biomechanics, Paracelsus Medical University, Salzburg, Austria

**Keywords:** Total knee arthroplasty, Periprosthetic joint infection, Gastrocnemius flap, DAIR, Joint arthroplasty, Systematic review, Acute infection, Implant retention, Soft tissue reconstruction

## Abstract

**Background:**

Acute periprosthetic joint infection (PJI) represents a major cause of early failure following total knee arthroplasty (TKA). In selected cases, implant retention strategies such as debridement, antibiotics, and implant retention (DAIR), including modified techniques such as debridement, antibiotic pearls, and retention of the implant (DAPRI), may be considered. However, the success of these approaches is strongly influenced by the condition of the surrounding soft tissues. In the presence of compromised or tenuous wound conditions, soft tissue reconstruction may play a critical adjunctive role. This review aims to evaluate the role of the gastrocnemius flap as an adjunct to implant retention strategies in the management of acute PJI following TKA.

**Methods:**

A systematic review was conducted in accordance with PRISMA guidelines. After screening 27 studies, five retrospective case series met the inclusion criteria, comprising a total of 73 patients with a mean follow-up of 48.7 months.

**Results:**

The medial gastrocnemius flap was the most used technique, accounting for approximately 70% of cases. Infection clearance rates ranged from 66% to 77%, while prosthesis retention was achieved in nearly 79% of patients. Gastrocnemius flap coverage was applied in conjunction with different PJI treatment strategies, including staged revision procedures and implant retention approaches. Early flap application, particularly when combined with debridement and implant retention in the presence of compromised soft tissues, was associated with improved wound healing and limb salvage, although the level of evidence remains limited.

**Conclusions:**

Gastrocnemius flap reconstruction appears to be a useful adjunct in selected patients undergoing implant retention strategies for acute PJI following TKA, particularly in the presence of compromised soft tissue conditions. Nevertheless, the current evidence is based on low-level, non-comparative studies, and definitive conclusions regarding its routine or preventive use cannot be drawn. Further prospective and comparative studies are required to better define indications, timing, and expected outcomes.

## Introduction

 Total knee arthroplasty (TKA) is one of the most successful procedures in orthopedic surgery, yet nearly 20% of patients remain unsatisfied with their clinical outcomes. Principal causes of TKA failure include periprosthetic joint infection (PJI), instability, failure of osteointegration, and polyethylene wear [1]. TKA failures can occur early (< 2 years) or late (> 2 years), and PJI represents one of the main causes of early complications [2]. PJI can have a devastating effect both locally and systemically, often leading to a decrease in daily activity level and an increase in mortality. While the incidence of PJI following primary TKA is approximately 1–2% over the lifetime of a prosthetic joint, nearly 25% of all TKA failures today are due to infection [2, 3].

Several surgical strategies have been proposed for the management of PJI, depending on the timing and chronicity of the infection, the causative microorganism, patient comorbidities, implant stability, and local soft tissue conditions [6, 7]. Exchange arthroplasty remains the standard of care in chronic and delayed PJIs, either as a one-stage or two-stage procedure, depending on microorganism virulence, patient-related factors, and local tissue status [8–11]. In contrast, for acute postoperative or early hematogenous infections, typically occurring within 4–6 weeks from surgery or within 7 days from symptom onset, debridement, antibiotics, and implant retention (DAIR) is commonly employed. A recent modification of the DAIR procedure, such as the DAPRI (Debridement, Antibiotic Pearls, and Retention of the Implant) technique, has shown promising results in selected acute cases by combining mechanical biofilm disruption with local antibiotic delivery through calcium sulfate beads [12]. Regardless of the selected surgical strategy, the integrity and quality of the surrounding soft tissue envelope play a pivotal role in determining treatment success. DAIR and DAPRI procedures, as well as revision arthroplasty, may be limited by inadequate soft tissue coverage, wound healing problems, or persistent infection, particularly in patients with compromised local tissue conditions [13]. Recent evidence also suggests that some commonly used perioperative measures, including intra-articular suction drains, may negatively influence outcomes by increasing blood loss and the risk of complications, thereby impacting soft tissue healing and infection control [11].

When direct wound closure is not feasible due to skin defects, excessive tension, or exposure of bone or prosthetic components, soft tissue reconstruction becomes essential and often requires plastic surgery expertise. Even in the absence of a true skin defect, excessive tension during wound closure in TKA may negatively affect healing and may benefit from additional coverage procedures. These interventions improve local oxygenation, facilitate antibiotic delivery, and modulate the immune response in infected joints [14].

Among reconstructive options, muscle flaps represent the preferred solution for achieving stable and durable coverage in infected total knee arthroplasty. Skin grafts are generally unsuitable due to their limited mechanical resistance, while fasciocutaneous flaps may provide insufficient vascularity in the presence of infection. In contrast, muscle flaps offer superior perfusion, adaptability, and resistance to infection [14]. In cases of medium-sized defects (approximately 4–6 cm) in the patellar or infrapatellar region with bone or implant exposure, a medial or lateral gastrocnemius flap is commonly employed as a reliable reconstructive option [14].

The gastrocnemius muscle flap, particularly its medial head, has become the most widely used flap for soft tissue reconstruction around the knee due to its robust vascular anatomy, proximity to the joint, and favorable arc of rotation. Its application has been reported across a broad spectrum of PJI treatment strategies, including implant retention procedures, single-stage and two-stage revision arthroplasty, and limb salvage situations. In addition to its established role in cases of overt soft tissue defects or prosthetic exposure, the gastrocnemius flap has also been proposed as an adjunct in selected high-risk cases, such as DAIR procedures performed in the presence of tenuous or compromised wound conditions. However, the evidence supporting early or preventive flap use in the absence of clear soft tissue defects remains limited and is primarily based on retrospective series.

This review aims to examine the role of the gastrocnemius flap in the treatment of periprosthetic joint infection following total knee arthroplasty, summarizing current evidence on indications, surgical techniques, outcomes, and complications across different PJI treatment strategies. Particular attention is given to its use both in implant retention approaches, such as DAIR, and in more complex reconstructive scenarios associated with chronic or recurrent infections, while acknowledging the limitations of the available evidence.

## Materials and methods

The review followed PRISMA (Preferred Reporting Items for Systematic Reviews and Meta-Analyses) guidelines [15], ensuring a thorough and systematic approach to data collection and analysis. This systematic review has also been registered with the International Prospective Register of Systematic Reviews (PROSPERO), under registration number 1,108,996.

### Search strategy

The search was performed across several online databases, including PubMed, Scopus, and Web of Science. The literature search covered all records from database inception to June 30, 2025, and no further update was performed prior to manuscript submission. The search string used was as follows: ((DAIR) OR (DAPRI) OR (PJI) OR (periprosthetic joint infection) OR (prosthetic joint infection)) AND ((flap) OR (gastrocnemius)). All retrieved records were imported into reference management software for duplicate removal.

We carefully examined the titles and abstracts of all retrieved articles to assess their eligibility for inclusion in the review. The criteria for inclusion were as follows: the studies must involve human adults, be published in English, and have publication dates up to June 2025. We included randomized trials, uncontrolled comparative trials, and case series. When there was uncertainty, the full article was retrieved for further examination. The senior author and the content area experts then obtained the full text of all articles and reviewed them to minimize any bias that could arise from preconceived opinions about the studies and their findings. This process was further enhanced by following up on the reference lists of relevant studies to identify additional articles. Two authors (D.G. and C.S.) independently reviewed the abstracts, obtaining the full texts for any abstracts that were inconclusive. Any differences between the reviewers were discussed, and if disagreements remained, the senior author (R.V. or G.M.) was consulted. The reference lists of the selected articles were manually checked to identify additional relevant studies. All selected studies were then analyzed retrospectively by three authors (D.G, A.E.M., C.S. and G.B.), who extracted and entered the data into an Excel worksheet. Finally, the data sheet was reviewed by four authors (R.V., D.G., C.S. and G.B.), who reached consensus on the extracted data. Additionally, the references of the identified papers were searched to find further relevant articles, and all journals were considered.

### Inclusion and exclusion criteria

**Fig. 1:**
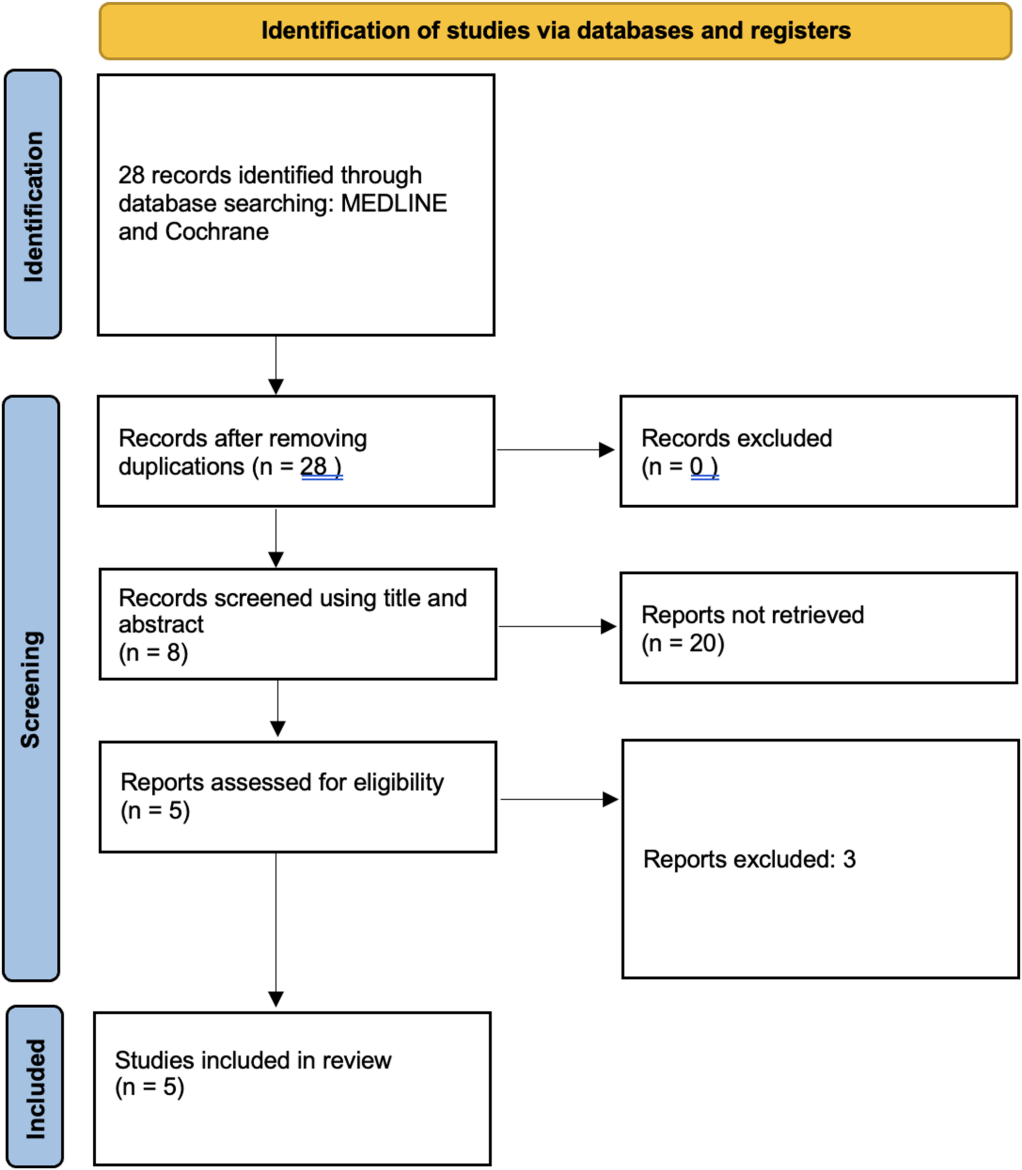
PRISMA 2020 flow diagram

The eligibility criteria for our analysis were established to include studies that met high methodological and reporting standards in the evaluation of the management of PJI. We included therapeutic clinical studies that specifically evaluated PJI which have been treated by gastrocnemius flap concomitant with the DAIR/DAPRI procedure, with a minimum follow-up period of 12 months for all patients. Eligible studies were required to be published in peer-reviewed journals, and be written in English. Furthermore, only studies with a full-text version available were considered. To maintain the quality and clinical relevance of our analysis, we excluded certain types of studies. These included review articles, case reports, technique articles, cadaveric studies, animal studies, and in vivo basic science research. Additionally, studies that did not provide outcome scores or lacked sufficient follow-up data were excluded (Table 1). Three reviewers (G.C., *A.E.M*., G.B. and R.T) independently conducted the screening and review process, evaluating the full texts of the selected articles to assess their eligibility and extract relevant data. In cases of uncertainty regarding study inclusion, the final decision was made by the senior author. Additionally, three authors (G.C., G.B., A.EM. and R.T) independently assessed the risk of bias using standardized evaluation criteria. Any disagreements were resolved through discussion, and when needed, a supervising author (R.V.) was consulted to reach consensus. A total of 27 articles were initially identified through database searching. Of them, 20 were excluded. The remaining 7 full-text articles were assessed for eligibility. Two studies were excluded based on the inclusion and exclusion criteria. As a result, 5 studies were included in the final qualitative synthesis. The study selection process is summarized in the PRISMA flow diagram (Fig. 1).

### Data extraction and analysis

The titles and abstracts were independently screened by two reviewers, R.M.C. and D.G. For abstracts that either met the inclusion criteria or caused uncertainty, full-text articles were obtained. These full texts were subsequently re-evaluated by the same two independent reviewers. Any discrepancies were resolved through assessment by the senior author, R.V. The methodological quality of each study was assessed using the Methodological Index for Non-Randomized Studies (MINORS) score, which offers a maximum of 24 points for comparative studies and 16 points for non-comparative studies. Two authors, R.V. and G.B., independently assigned MINORS scores and reached consensus on the final score. Given the substantial clinical and methodological heterogeneity among the included studies, particularly regarding surgical indications, timing of flap application, outcome definitions, and follow-up duration, a quantitative meta-analysis was not feasible. Therefore, a qualitative narrative synthesis was performed. Descriptive statistics were used to summarize the data. Continuous variables are reported as means with standard deviations when available, while categorical variables are presented as frequencies and percentages. No inferential statistical analyses were performed. The gathered data were analyzed and organized using SPSS 26 software (SPSS, Inc., Chicago, IL, USA).

### Extracted variables and outcomes

From each included study, we extracted data on demographic and clinical variables, surgical techniques, outcomes, and complications. Demographic variables included patient age, sex and comorbidities while clinical variables included type of pathogen and flap (medial/lateral gastrocnemius flap). Although antibiotic treatment was considered a relevant variable, it was not consistently reported across studies and was therefore excluded from the analysis. We also recorded revision surgeries prior to the gastrocnemius flap procedure. The primary clinical outcome of interest was infection clearance, together with flap healing, as well as prosthesis salvage.

Complications were extracted as reported in each study. Since the original articles did not consistently differentiate between major and minor complications, we reported all adverse events without subclassification.

However, most included studies did not provide formal estimates of relative effect, such as risk ratios or hazard ratios. Given the heterogeneity in study design and outcome reporting, we performed a qualitative synthesis of the data.

## Results

### Patient demographics

A total of five studies were included in the final qualitative synthesis, comprising 73 patients. The mean patient age across the included studies was 69.1 ± 1.9 years, with reported mean ages ranging from 67 to 71.8 years. Standard deviations for age were inconsistently reported in the original studies and could not be extracted uniformly. Sex distribution was reported in all five studies, with 29 male patients (39.7%) and 44 female patients (60.3%) (Table 2). The mean follow-up duration was 48.7 ± 23.1 months, ranging from 27 to 96 months. Measures of dispersion for follow-up time were variably reported and therefore could not be consistently summarized across studies. Overall, 31 of 73 patients (42.5%) underwent DAIR or DAPRI procedures, while the remaining patients were managed with staged revision strategies. According to the modified Coleman Methodology Score (mCMS), the mean methodological quality score of the included studies was 59 points (range 44–73) (Table 1).

### Comorbidities

Comorbidity data were explicitly reported in three of the five included studies, accounting for 43 out of 73 patients (58.9%). This incomplete reporting represents a relevant source of potential bias, as comorbid conditions are known to significantly influence infection control and reconstructive outcomes. In the study by Kim et al., which included 13 patients, the following comorbid conditions were documented: diabetes mellitus (*n* = 4), smoking (*n* = 5), obesity (*n* = 3), peripheral vascular disease (*n* = 2), and rheumatoid arthritis (*n* = 1).

Young et al. reported comorbidities in all 17 patients in their cohort. The conditions included hypertension, chronic kidney disease (CKD), atrial fibrillation (AF), diabetes mellitus (DM), rheumatoid arthritis (RA), coronary artery bypass grafting (CABG), cerebrovascular events (CVA), and venous thromboembolism (VTE). The average number of prior procedures before referral was 2 per patient (range 0–5). McCulloch et al. did not list individual comorbidities but reported that 20 of 30 patients (66.7%) had undergone at least one prior revision procedure, and 17 of 27 patients (63%) had previously failed a revision for periprosthetic joint infection. The studies by Boadas-Gironès et al. and Müller SLC et al. did not report comorbidity data.

### Pathogen types

Microbiological findings were described in four of the five included studies, revealing a heterogeneous spectrum of causative organisms in periprosthetic joint infections (PJIs) managed with flap coverage.

In the study by Boadas-Gironès et al., microbiological cultures were positive in 9 out of 12 patients. Identified pathogens included Staphylococcus epidermidis in one case, Escherichia coli in one case, Enterococcus species in two cases, Pseudomonas aeruginosa in one case, and Bacillus cereus in one case. Three patients had polymicrobial infections, while cultures remained negative in three cases. McCulloch et al. reported intraoperative culture results for all 30 patients. A polymicrobial profile was observed in 15 patients. The remaining isolates included Staphylococcus aureus in 15 cases, Staphylococcus epidermidis in 5, Candida species in 3, Lactobacillus species in one case, and Rothia kristinae in one case. Two patients had negative cultures. In the study by Kim et al., infections were attributed to methicillin-resistant Staphylococcus aureus (MRSA) and coagulase-negative staphylococci (CoNS). However, the specific number of cases per organism was not detailed. Müller SLC reported a single case of periprosthetic joint infection with negative microbiological culture. Young et al. did not report any microbiological data. Culture-negative infections were also reported, highlighting the variability of microbiological diagnosis across studies.

### Surgical technique and type of flap

Across the analyzed studies, a total of 66 muscle flaps were performed. The medial head of the gastrocnemius was by far the most commonly used flap, representing approximately 70% of all cases. The lateral head of the gastrocnemius was utilized in about 11% of patients, while a combined use of both medial and lateral heads was reported in roughly 5% of cases. Other flap types, such as the anterior tibialis flap and the laterally based split-anterior portion (LSAP) of the gastrocnemius, were reported but were rare, accounting for about 1.5% each. In some cases, approximately 11%, the flap type was not specified. Early flap application was defined as gastrocnemius flap coverage performed during the initial surgical management of infection, including concomitant DAIR/DAPRI procedures or during the first stage of revision arthroplasty following PJI diagnosis. Late flap application referred to flap coverage performed after failed prior surgical attempts or during subsequent revision stages.

Looking at individual studies, McCulloch et al. reported 30 cases where the medial gastrocnemius flap was used in 80% of patients, the lateral head in 13%, and a combined flap in nearly 7%. Kim et al. described 13 cases, with a majority (77%) receiving the medial gastrocnemius flap, 15% receiving the lateral head flap, and one patient receiving an anterior tibialis flap. Boadas-Gironès et al. exclusively used the medial gastrocnemius flap in all 12 of their patients. Müller SLC et al. reported a single case employing the laterally based split-anterior portion of the gastrocnemius. Finally, Young et al. described 15 cases receiving the gastrocnemius flap.

### Flap complications

Flap-related complications were reported in 15 out of 73 patients (20.5%). The most common complications were flap necrosis and infection, each occurring in about 8% of cases. Partial flap necrosis was noted in 4 patients (5.5%), while total flap failure was rare, observed in only 1 patient (1.4%). Infections at the flap or donor site affected 6 patients (8.2%). Other complications included wound dehiscence in 3 patients (4.1%) and hematoma formation in 2 patients (2.7%). Vascular thrombosis and significant donor site morbidity were not reported. Management of these complications varied according to severity. Partial flap necrosis was generally treated with local wound care and, in some cases, minor surgical debridement. The single case of total flap failure required flap revision and a secondary reconstructive procedure. Infections were managed with targeted antibiotic therapy and, when necessary, surgical drainage or debridement. Wound dehiscence cases were treated conservatively with dressings, with one case necessitating minor surgical intervention.

For instance, McCulloch et al. reported 6 complications in 30 patients (20%), predominantly minor necrosis resolved with conservative measures. Kim et al. observed 3 complications (23%), including one total flap failure requiring revision surgery and two infections treated with antibiotics and drainage. Boadas-Gironès et al. documented a single wound healing complication (8%) successfully managed with local care. Müller SLC et al. and Young et al. did not report flap-related complications.

### Clinical outcomes

Infection clearance was achieved in at least 55 of 73 patients (75.3%), based on explicit reporting from the included studies. Clinical outcomes varied depending on the surgical strategy and timing of flap application, although direct comparisons were not possible due to the uncontrolled nature of the data.

In the study by Boadas-Gironès et al., 8 out of 12 patients (66.7%) achieved infection resolution, 2 required a two-stage revision, 1 underwent amputation, and 1 was managed with suppressive antibiotic therapy. The authors noted a higher success rate in patients undergoing combined DAIR and flap coverage.

Kim et al. reported that 9 of 13 patients (69.2%) had infection control with flap preservation, while 3 experienced recurrent infection and 1 required amputation after a prolonged course.

In McCulloch et al., clinical outcomes included 23 cases of limb salvage (76.7%), with 7 patients (23.3%) requiring further interventions such as amputation (*n* = 5), long-term suppressive therapy (*n* = 9, some overlapping), or additional surgeries.

The single patient in Müller SLC’s case report achieved complete infection clearance, with knee flexion of 110°, strength graded M5, and an AKSS score improvement from 97/50 to 99/100 postoperatively.

Young et al. did not provide detailed data on infection resolution or clinical scoring outcomes but noted multiple interventions prior to definitive soft tissue coverage. Across the studies, amputation was reported in a total of 5 patients (33.3%), while functional scores, where available, showed preserved or improved joint function in patients with successful flap and implant retention.

### Implant survival

Data on implant survival were available for 62 patients. Prosthesis retention was reported in 49 patients (79.0%) at final follow-up, while implant loss occurred in 13 patients (21.0%) due to persistent infection, mechanical failure, or amputation. Reported prosthesis retention rates ranged from 66% to 77% across studies, with variability in follow-up duration and patient characteristics.

In the study by Boadas-Gironès et al., prosthesis retention was achieved in 8 out of 12 patients (66.7%). Two patients required a two-stage revision, one underwent amputation, and one was managed with long-term suppressive antibiotic therapy.

Kim et al. reported implant survival in 9 of 13 patients (69%), while 3 patients required further procedures and 1 ultimately underwent amputation after 5 years.

In the cohort analyzed by McCulloch et al., 23 of 30 patients (76.7%) maintained their prosthesis at follow-up. The remaining 7 patients experienced failure, including 5 amputations and 2 cases requiring prolonged suppressive treatment or re-revision.

Müller SLC reported a single case in which the implant was successfully preserved and infection resolved.

Young et al. did not report implant survival directly, but mentioned multiple procedures prior to referral, and no clear data regarding definitive prosthesis status were provided.

Overall, the data indicate that implant survival following muscle flap coverage in infected total knee arthroplasty ranged from 66% to 77% across studies, with variable follow-up durations. In most cases where the prosthesis was retained, infection was controlled, and limb function was preserved.

**Table 1:** Overview of demographics

Author	Year	Type of Study	MCMS	*N*° patients	Mean age (years)	Gender (M/F)	Mean Follow-up period (months)	Comorbidities
Boadas-Gironès et al. [14]	2024	retrospective study	44	12	71,8	4:8	96	-
Müller SLC et al. [42]	2024	retrospective study	57	1	71	0:1	27	-
Kim BI et al.[43]	2024	retrospective study	73	13	67	5:8	36,73	Smoking; Diabetes; Obesity; Peripheral vascular disorder; Rheumatoid arthritis
McCulloch RA et al. [36]	2021	retrospective study	63	30	68,9	12:18	50,94	-
Young K et al. [40]	2016	retrospective study	58	15	69	8:9	33	Hypertension; chronic kidney disease, atrial fibrillation; Diabetes mellitus; Rheumatoid arthritis; Coronary artery bypass graft; Cerebrovascular event; Venous thromboembolism
Total	-	-	-	71	69,08	29:44	21,44

**Table 2:** Surgical features and previous revision surgeries

Author	*N*° patients	Flap technique	Flap timing	Prior revision surgeries
Boadas-Gironèet al. [14]	12	12 Medial gastrocnemius flap	-	-
Müller SLC et al. [42]	1	1 Pedicled LSAP gastrocnemius	-	-
Kim BI et al.[43]	13	10 Medial gastrocnemius; 2 Lateral gastrocnemius; 1 Anterior tibialis;	-	-
McCulloch RA et al.[36]	30	80% (24 patients) isolated medial gastrocnemius flap; 13% (*n* = 4) isolated lateral gastrocnemius flap; 6% (*n* = 2) both lateral and medial gastrocnemius flaps	21 first-stage revision; 4 debridement and implant retention; 4 single stage; 1 s stage;	20 of 30 (67%) patients had previously undergone at least one revision surgery; 63% (17 of 27 patients) had previously failed a revision procedure for periprosthetic joint infections.
Young K et al. [40]	15	-	-	Mean number of procedures post-arthroplasty prior to transfer was 2 (range 0–5)

**Table 3:** Outcomes and complications

Author	Flap complications	Saved prosthesis (Yes/No)	Clinical outcome
Boadas-Gironèet al. [14]	-	8 Yes	8 infection clearance; 2 required a two-stage revision; 1 needed amputation 1 received a suppressive treatment to infection control
Müller SLC et al. [42]	Partial flap necrosis; Small wound healing disorder;	-	-
Kim BI et al. [43]	flap revision, 3 focal necrosis; 3 repeat stsg; 1 wound dehiscence;	9 Yes	3 spacer; 1 amputation (after 5 years)
McCulloch RA et al. [36]	-	23 Yes	23 salvage 8 further surgery procedures exclud- ing amputation; 9 Long-term antibiotic suppression; 7 Amputation;
Young K et al. [40]	-	-	3 amputation 12 salvage

## Discussion

Periprosthetic joint infection is one of the main causes of total knee arthroplasty (TKA) failure, along with instability and aseptic loosening of the implant [16]. PJI represents one of the principal causes of early revision of TKA, affecting patients’ quality of life [17] while increasing health costs [18]. The infection rate following primary knee or hip replacement is estimated to be between 0.3 and 1.9% [19], while the average PJI rate of occurrence within the first six months after surgery was 4.2–4.8% [20]. Periprosthetic joint infections (PJIs) are typically classified into three main categories based on their timing. Early infections, arising within the first 4 to 6 weeks post-surgery; delayed infections tend to develop between 3 and 24 months after the procedure and late infections, which manifest more than 2 years after the initial surgery [21]. Revision arthroplasty after PJI remains challenging, particularly in the presence of soft tissue defects, with high rates of failure reported [22–25].

In cases of chronic or late-stage infections, implant revision is generally recommended. This can be performed either as a single-stage or two-stage procedure. Conversely, for early postoperative infections or acute haematogenous infections diagnosed within 7 days of symptom onset, the preferred approach is often DAIR (Debridement, Antibiotics, and Implant Retention). This method is considered appropriate, particularly when the prosthesis is stable, well-fixed, and there is no presence of a sinus tract [27]. More recently, DAPRI (debridement, antibiotic pearls, and retention of the implant) has emerged as a modified implant retention strategy in selected early PJIs, showing encouraging outcomes compared to standard DAIR in retrospective series [12, 28].

Regardless of the surgical strategy adopted, the success of PJI treatment is strongly influenced by the condition of the surrounding soft tissues. In many patients, particularly those with previous revision or trauma, soft tissue defects or poor wound healing capacity are an obstacle to healing, and correlate with very high failure rates in revision TKAs with high rates of amputation, reinfection and mortality (30,31,32,33). In the presence of exposed prosthetic material, bone, or necrotic tissue, or when direct closure risks excessive tension or wound breakdown, soft tissue reconstruction becomes an essential component of treatment rather than a secondary consideration. Skin grafts provide unstable coverage [32]. The fascio-cutaneous flap is not vascularized enough [33]. Free flaps are a good choice, but they may lead to a high risk of flap loss in the case of very short pedicles and very deep recipient vessels [32]. The ideal flaps are the muscle ones. The main advantage of muscle flaps over fascio-cutaneous flaps is the remodeling of the flap over time, as the latter changes color, and has a lower vascularity and strength index compared to muscle flaps [32].

The gastrocnemius muscle flap, in particular its medial head, is the most commonly used option in this context due to its vascular anatomy and arc of rotation [32, 34]. It is mainly used for medium and large soft tissue defects around the knee. Beyond its reconstructive role, flap coverage contributes to infection control by improving local perfusion and facilitating antibiotic penetration [35, 36]. Consequently, gastrocnemius flap coverage has been widely applied in conjunction with different PJI treatment strategies, including implant retention procedures, staged revision arthroplasty, and salvage situations [14, 37].

The timing of flap application represents a key variable in PJI management, although the optimal timing remains a matter of debate. Several authors have reported favorable outcomes when gastrocnemius flaps were applied early, either during the acute phase of infection or at the first stage of revision. Theil et al. demonstrated that early gastrocnemius flap use facilitated durable soft tissue coverage and reduced the risk of recurrent infection and reoperation [34]. Similarly, Corten et al. reported their experience in 30 patients, showing that in 70% of cases the flap was applied during the first stage of a two-stage revision, while in the remaining patients it was performed either in the context of DAIR procedures or during single-stage revisions [37]. These findings suggest that flap reconstruction can be successfully integrated into different PJI treatment strategies, particularly when performed before the development of severe chronic soft tissue compromise. Technique-wise, the medial gastrocnemius flap was most frequently used, followed by lateral and combined flaps, depending on defect location and extent [14, 37].

Literature supports the role of medial gastrocnemius flaps in achieving infection control and implant salvage. The reported implant survivorship varies between 48% and 92% [38].

Successful results have been reported with medial gastrocnemius flap reconstruction both when performed at the first stage of revision (explantation and antibiotic spacer implantation) and at the second stage (reimplantation) [38, 39]. Evidence suggests, however, that the timing of flap coverage may influence the final outcome depending on the local tissue condition. In patients with frank soft-tissue defects, early flap application appears to be advantageous, as it provides immediate stable coverage, enhances antibiotic penetration, and reduces the risk of persistent infection. Corten et al. reported 100% flap survival and limb salvage in 77% of cases, despite 97% of patients presenting multiple local compromising factors [37], supporting the role of the flap as a key element in limb preservation. Similarly, Young et al. observed high success rates when flaps were used early to secure implant coverage and promote wound healing [40]. More recently, Boadas-Gironès et al. demonstrated that combining DAIR with gastrocnemius flap reconstruction improved infection control even when PJI was only suspected and not yet confirmed, highlighting that early flap use can be beneficial also in “borderline” situations [14]. This observation raises the hypothesis that flap coverage might be beneficial in selected “borderline” cases with tenuous soft tissues; however, robust comparative evidence supporting preventive or routine early flap use in the absence of clear defects is currently lacking. Therefore, such an approach should be considered exploratory and limited to carefully selected patients.

Despite the above-mentioned advantages, post-procedural complications such as flap necrosis, wound dehiscence and delayed healing have been reported, albeit in relatively low rates. Comorbidities such as diabetes, peripheral vascular disease and previous revisions significantly influence outcomes, as well as tobacco use and growth of multiple organisms [14, 23, 40]. These risk factors contribute to delayed healing, increased re-infection rates and increased risk of amputation.

The results of our review are consistent with the literature on the management of PJI requiring additional soft tissue reconstruction. Reported infection clearance rates of 70% to 90% and prosthesis retention above 80% are in line with those described in several series. However, it is well established that the presence of prosthesis exposure or significant soft tissue compromise is associated with worse outcomes if not addressed adequately [36, 40]. In this setting, early gastrocnemius flap coverage has been shown to mitigate the negative prognostic impact of exposure, providing a vascularized and durable envelope that enhances local antibiotic activity and reduces reinfection risk.

For example, McCulloch et al. reported a 77% limb salvage rate despite most patients presenting multiple local and systemic risk factors, confirming that flap reconstruction can achieve outcomes comparable to those seen in PJI patients without initial exposure when performed in a timely fashion [36]. Similarly, Young et al. observed that early flap use in the presence of exposure led to prosthesis retention rates overlapping with those of non-exposed cases [40]. These data suggest that, while patients with frank defects are at higher baseline risk, prompt flap application, ideally at the first surgical stage or concomitant with DAIR when feasible, can substantially reduce this gap.

Thus, the timing and indication for flap use are central: in patients with overt tissue loss, immediate reconstruction appears to “neutralize” the poor prognosis otherwise associated with exposure; in patients without overt exposure but with tenuous soft tissues, a preventive flap may also improve outcomes, although comparative evidence remains limited.

Similarly, Boadas-Gironès et al. demonstrated that the combination of gastrocnemius flap and DAIR resulted in higher rates of infection control compared with flap coverage alone, with success rates of 66.6% versus 33.3%, respectively [14]. Furthermore, our results confirm the functional improvements observed in studies that used objective clinical metrics. For example, Müller SLC et al. observed improvements in both clinical and functional AKSS scores following combined treatment with DAIR and flap reconstruction [42].

While these outcomes are encouraging, it is important to note that some studies also emphasize a persistent discrepancy between clinical success and patient-reported outcomes. As highlighted by Abosala and Ali, even when infection was eradicated and limb salvage achieved, quality of life and functional capacity often remained limited, particularly in patients with a high burden of comorbidities or multiple prior revisions [23, 37, 40, 41].

This underscores the importance of careful patient selection, realistic expectations, and individualized treatment planning. The complexity of these cases highlights the role of a multidisciplinary approach involving orthopedic surgeons, plastic surgeons, infectious disease specialists and wound care teams. A collaborative model ensures optimal timing, techniques and postoperative care, minimising morbidity and improving outcomes. However, studies have shown the advantage of having experienced orthopaedic surgeons perform both debridement and flap reconstruction, reducing the burden of resource use and maintaining efficacy [36, 39].

Several limitations must be recognised. Firstly, the studies included in this review are predominantly retrospective case series. The lack of randomised controlled trials (RCTs) is mainly due to ethical and logistical reasons in surgically complex and acutely ill populations. Secondly, the included studies had considerable heterogeneity with regard to surgical indications, pathogen types, flap timing and outcome measures. Functional scores were inconsistently reported and only a few studies used standardised parameters. Thirdly, the duration of follow-up varied widely, from short-term (12 months) to long-term (up to 96 months), which complicates the comparison of prosthesis survival and long-term infection control. Finally, it is necessary to consider publication bias, as studies reporting positive outcomes may be more likely to be published. The relatively small number of patients in each study further limits the possibility of generalisation of results. Furthermore, none of the reviewed studies reported in detail the specific indications for the use of the gastrocnemius flap. This limitation makes it difficult to objectively assess the outcomes, since the flap was applied in heterogeneous clinical contexts without a clear reason, other than the generic presence of soft-tissue defects. This highlights the need for shared guidelines that clearly define the indications for its use in different clinical scenarios.

Despite these limitations, this review consolidates current evidence supporting the gastrocnemius flap as a valuable adjunct in the management of PJI following TKA, particularly in cases with compromised soft tissues.

## Conclusion

Periprosthetic joint infection following total knee arthroplasty remains a complex clinical problem, particularly in the presence of compromised soft tissues. Effective soft tissue management is a key component of successful PJI treatment across different surgical strategies, including implant retention and staged revision procedures. Based on the available literature, gastrocnemius flap reconstruction appears to be a useful adjunct in selected cases of PJI, contributing to durable coverage, limb salvage, and infection control, especially in the presence of overt soft tissue defects or prosthetic exposure. Its use has been reported across different PJI treatment strategies, including DAIR, DAPRI, and revision arthroplasty. The potential role of early or preventive gastrocnemius flap application in implant retention procedures remains supported by limited, retrospective evidence and should be considered hypothesis-generating rather than evidence-based. Consequently, definitive conclusions regarding optimal timing and patient selection cannot be drawn, and further prospective comparative studies are warranted.

## Data Availability

All of the data we analyzed and the tables we compiled are available for any clarification.
